# Music in Research and Rehabilitation of Disorders of Consciousness: Psychological and Neurophysiological Foundations

**DOI:** 10.3389/fpsyg.2015.01763

**Published:** 2015-11-27

**Authors:** Boris Kotchoubey, Yuri G. Pavlov, Boris Kleber

**Affiliations:** ^1^Institute for Medical Psychology and Behavioural Neurobiology, University of Tübingen, Tübingen, Germany; ^2^Department of Psychology, Ural Federal University, Yekaterinburg, Russia

**Keywords:** consciousness, DoC, music, rehabilitation, psychology, neurophysiology

## Abstract

According to a prevailing view, the visual system works by dissecting stimuli into primitives, whereas the auditory system processes simple and complex stimuli with their corresponding features in parallel. This makes musical stimulation particularly suitable for patients with disorders of consciousness (DoC), because the processing pathways related to complex stimulus features can be preserved even when those related to simple features are no longer available. An additional factor speaking in favor of musical stimulation in DoC is the low efficiency of visual stimulation due to prevalent maladies of vision or gaze fixation in DoC patients. Hearing disorders, in contrast, are much less frequent in DoC, which allows us to use auditory stimulation at various levels of complexity. The current paper overviews empirical data concerning the four main domains of brain functioning in DoC patients that musical stimulation can address: perception (e.g., pitch, timbre, and harmony), cognition (e.g., musical syntax and meaning), emotions, and motor functions. Music can approach basic levels of patients’ self-consciousness, which may even exist when all higher-level cognitions are lost, whereas music induced emotions and rhythmic stimulation can affect the dopaminergic reward-system and activity in the motor system respectively, thus serving as a starting point for rehabilitation.

## Introduction

The aim of the present paper is to show that music is a particular kind of auditory stimulation that may be most beneficial for use in patients with disorders of consciousness (DoC) in both research and therapy. With respect to therapy, the enormous complexity of such studies partly accounts for the currently low number of well-controlled trials and hence the limited demonstration of evidence-based effects of music therapy in DoC (see Giacino et al., 2012). However, one-time experimental interventions using musical stimuli yielded promising results in a few studies with middle-sized DoC samples (e.g., Formisano et al., 2001; O’Kelly and Magee, 2013; Magee and O’Kelly, 2015). Less clear-cut are the data of music therapy interventions, which are summarized in Table 1. As can be seen in the table, only three studies (Formisano et al., 2001; Raglio et al., 2014; Sun and Chen, 2015) tested the effects of musical therapy using 10 or more DoC patients. Only the last one employed a sufficient level of control and showed some promising results. However, these data are in need of replication.

**TABLE 1:** **Music therapeutic interventions and outcomes in DoC**.

**Source**	**Participants**	**Design**	**Outcome**
Formisano et al. (2001)	Thirty-four MCS patients, 13–70 years, *M* = 35.94; 18 TBI, 16 non-TBI	Music therapy program included singing or playing different musical instruments.Three 20–40 min sessions per week during 2 months.	Decreasing in inertia or psychomotor agitation in 21 patients.No significant change of CRS scores.
Magee (2005)	One VS patient, >50 years old anoxic brain injury	Music therapy program with singing and playing musical pieces. Music selection based on the participant’s life history.No information about the duration of the program.	The patient demonstrated some behavioral responses in response to music and song exposition.No information about changes in objective measures.
Raglio et al. (2014)	Four MCS and six VS patients (five with anoxic brain injury, four hemorrhage, one TBI)	Music therapy included two cycles of 15 sessions (three sessions/week, 30 min each). The cycles spaced out by 2 weeks.	Improvements of some observed behaviors in MCS patients: eye contacts, smiles, communicative use of instruments/voice, reduction of annoyance, and suffering expressions. VS patients only increased eye contacts.
Seibert et al. (2000)	One MCS patient, 20 years old after severe hypothermia, cardiac arrest, and brain anoxia; GCS score – 12Rancho Los Amigos Scale – 4	Music therapy program involved exposure to oboe music, physical contact with the instrument, and the presentation of favorite music during 2.5 years.	At the end of the program: GCS score – 15, Rancho Los Amigos Scale – 6; Persisting moderate deficits in orientation/attention, visual-spatial skills, memory, and language. Reading comprehension and ability to follow commands were at a moderate level.
Lee et al. (2011)	One VS patient, age 45 yearsIntracerebral hemorrhageGCS score – 4	ECG data collected during 7 weeks. First week: six baseline sessions with no music, each lasting for 180 min. Next 6 weeks: six music sessions when the patient listened to Mahler’s symphony no. 2, each session lasted for 210 min.	Changes in the standard deviation of time sequences showed positive changes in the cardiovascular system.
Steinhoff et al. (2015)	Four VS patients after cardiopulmonary resuscitation	Music therapy group (*n* = 2): standard care plus live and individual music therapy sessions for 5 weeks (three sessions/week, about 27 min each). Control group (*n* = 2): only standard care.PET in the baseline in rest state; PET at the end of the second and sixth weeks in response to musical stimulation (both in the music and control groups).	Patients in the music therapy group appeared to show higher brain activity than control group patients in the last PET scan.
Sun and Chen (2015)	Forty TBI coma patients, 18–55 years oldGCS score between 3 and 86.55 ± 2.82 days after injury	Music therapy group (*n* = 20): listening to their favorite and familiar music for 15–30 min three times every day during 4 weeks. Control group (*n* = 20): waiting control.	GCS scores increased significantly in both groups, yet significantly more in the music therapy group. Relative power of slow EEG rhythms decreased in both groups, yet these changes were significantly stronger in the music therapy group.

CRS, Coma Recovery Scale; ECG, electrocardiography; EEG, electroencephalography; GCS, Glasgow Coma Scale; MCS, minimally conscious state, PET, positron emission tomography; TBI, traumatic brain injury; VS, vegetative state.

In contrast to therapeutic effects in DoC, we can draw on a large number of studies that examined the highly specific effects of music on basic perceptual, higher-cognitive, and emotional processes in the brain of healthy subjects, and derive suggestions for their use in DoC. In this review, we will concentrate on features of music that play, or can play, a significant role in the examination and/or rehabilitation of chronic DoC. We do not present a comprehensive review on music perception and cognition but rather intend to analyze the potential and applicability of music stimulation in DoC.

This review starts with some fundamental reasons why auditory stimulation might be particularly useful in DoC. We then first provide essential information about the neural specializations of auditory processing (e.g., basic sensory and sensorimotor mechanisms) before describing higher-level perceptual organization of sound, including the neural differences associated with the processing of musical syntax and semantics. After we moved on to discuss the potential benefits of multisensory stimulation in DoC, we finally provide evidence and suggestions for the use of musical stimulation as a therapeutic tool with respected to effects on cognition, emotion, and stress in DoC. The scheme we adopted throughout all sections is to first describe how healthy subjects respond to music before reviewing the evidence-based practice or potential application of music stimulation in chronic DoC.

## Why Auditory Stimulation in DoC?

Many DoC patients cannot see. Andrews et al. (1996) indicated in their frequently cited article that blindness is a major issue contributing to the exceptionally high rate of misdiagnosis in DoC: “The very high prevalence of severe visual impairment… is an additional complicating factor since clinicians making the diagnosis of the vegetative state place great emphasis on the inability of the patient to visually track or blink to threat” (p. 15). Moreover, even if both sensory pathways from the retina to the visual cortex and the cortical centers themselves are intact, this does not indicate that a DoC patient can see, as the role of motor control in visual perception is vital. To perceive anything more than just light, not only must the eyelids be open but also the ocular muscles and their controlling brain areas must be able to perform following and searching saccadic movements, a skill that is drastically reduced in vegetative state (VS) and also severely impaired in minimally conscious state. Conversely, the ability to consistently perform following gaze movements is considered a criterion to rule out a DoC diagnosis, whereas inconsistent followings may be compatible with the diagnosis the minimally conscious state (MSC+; Bruno et al., 2011). In an unpublished pilot study, we examined electroencephalography (EEG) responses to visual stimuli as simple as checkerboard patterns in five patients who fulfilled the diagnostic criteria of MCS+ according to Bruno et al. (2011). We failed to record a consistent evoked potential (EP) in any of them, although EPs to simple flash as well as the primary EP complex (P1–N1–P2) to auditory stimuli were virtually normal.

The situation seems indeed to be completely different in the auditory modality, not only because ears cannot be physically closed like eyes but also because active voluntary control of peripheral muscles is not vital for immediate sound sensation, although motor and corresponding somatosensory factors are of great importance in the perception of complex auditory stimulation (see below). We could not find data about the prevalence of lacking brain stem auditory EPs (BSAEP) in DoC, perhaps because the presence of this response is an inclusion criterion in most studies and therefore patients without BSAEP would be excluded from the very beginning. It follows that studies in DoC should not only provide detailed exclusion criteria with respect to auditory EPs but also how many patients were effectively excluded from the sample based on these rules. In fact, auditory EPs are frequently used in ENT clinics to distinguish between normal or hearing-impaired states in otherwise healthy infants (Paulraj et al., 2015). Among 83 VS patients with at least partially preserved BSAEP, 71 patients (i.e., 86%) also showed cortical EP components (as a rule, N1). If we introduce a further criterion and eliminate 10 VS patients with large-amplitude diffuse delta waves dominating the EEG all the time, only two patients (2.7%) with BSAEP would not show cortical EPs. All 49 examined MCS patients exhibited cortical auditory EPs. A subsample of this patient group (i.e., 50 VS and 39 MCS patients) has been reported in detail elsewhere (Kotchoubey, 2005; Kotchoubey et al., 2005). Notably, we observed a highly significant N1 component to complex tonal stimuli and even highly differentiated responses to speech (Kotchoubey et al., 2014) in patients with anoxic brain injury up to 11 years in the VS with Level 4 brain atrophy according to the classification of Galton et al. (2001) and Bekinschtein et al. (2009). Moreover, about half of the DoC patients without a specific lesion of the right temporal lobe exhibited significant responses to affective prosody (exclamations like “wow,” “ooh,” etc.: Kotchoubey et al., 2009). Taken together, deafness does not seem to be a major problem in most DoC patients. If deafness should be present, however, it is usually detected at very early stages of the disease because BSAEP are routinely recorded from the very beginning in most German hospitals for neurological rehabilitation. The cases of cortical deafness in DoC seem to be rare. If, as suggested in a stepwise procedure (Kübler and Kotchoubey, 2007; Kotchoubey et al., 2013), we first exclude patients without brain stem EP and patients with diffuse delta activity (the two groups usually overlap strongly), cortical auditory EPs can be obtained in nearly every DoC patient. Therefore, we suggest to use complex tonal stimuli for auditory EPs as a rule and the use of music therapy only in DoC patients with preserved neurophysiological findings [e.g., brain-stem and middle-latency auditory EPs and event-related potential (ERP)].

## Neural Specializations for Auditory Processing

### Basic Considerations

The oscillatory structure of acoustic events can be conceptualized as two perceptually quite distinct components: one that consists of *higher frequencies*, which provide the basis of *pitch* and *timbre* perception, and one that consists of *lower frequencies*, which provide the basis of musical rhythm and meter perception (i.e., the temporal organization of sounds). According to a well justified (although not yet in all respects empirically tested) hypothesis, this distinction has been related to two discrete anatomical and physiological components of the auditory system that have been classically described in the neurophysiology of afferent systems as specific versus non-specific, or lemniscal versus extralemniscal subsystems (e.g., Abrams et al., 2011).

Anatomically, the auditory cortex is subdivided into the primary cortex, or A1 [Brodmann area (BA) 41], the belt, or A2 (BA 42), and the parabelt, or A3 (BA22). The belt extends from inside the lateral sulcus or the supratemporal plane out onto the open surface of the superior temporal gyrus (STG) and receives independent input from the superior colliculus separately from A1 (Pandya, 1995). Neurons in the ventral part of the medial geniculate body (MGB) terminate in deeper layers (mainly, Layer 4 and the deep portion of Layer 3) of A1 and their impulsion immediately elicits action potentials in pyramidal neurons located there. The narrow frequency tuning of these neurons results in a relatively tonotopic organization of A1 (Formisano et al., 2003), providing specific frequency information and thus contributing to the perception of pitch and timbre (the “content” of a melody). In contrast, neurons located in various parts of the MGB (mostly in its dorsal division) that target at superficial Layers 1 and 2 of A1 and the belt, are more broadly tuned and deliver non-specific information. Activating apical dendrites of the pyramidal cells, they do not directly result in their firing, but rather regulate the firing threshold by “warming up” pyramidal neurons according to the basic rhythm (or the metrical “context”) of a musical phrase. The high-frequency content is therefore synchronized with the low-frequency context in such a way that responses “driven” by events associated with contextual accents are amplified, while the responses that occur out of beat are weakened. The context is therefore created by a modulatory input, and the content by a “driving” input of the auditory cortex (Musacchia et al., 2014).

As regards pitch perception, Rauschecker (1997, 1999) and Rauschecker et al. (1997) was probably the first who demonstrated, in macaque monkey experiments, the independence of the processing of pure tones and chords. Since the primary auditory cortex (BA 41) and the belt (A42) receive largely independent input, the tonotopic structure that is typical for the superior colliculus and A1 is basically lost in the belt and even more so in the parabelt. Pure tones are therefore the least effective auditory stimulation to elicit neuronal responses in these areas (Rauschecker, 1997), which may have implications for their use in DoC. In contrast, the cells of the belt strongly respond to complex sounds and frequency-modulated sweeps, indicating the non-reductive processing of complex sounds that builds the basis for the perception of pitch modulation independently of intensity (Rauschecker, 1999). The same research team further hypothesized that the auditory system, like its visual counterpart, entails two different pathways to higher-order cortical areas, designed for processing spatial and temporal information, the “where” and “when” subsystems (Romanski et al., 1999). This hypothesis, however, remains under debate (e.g., Griffiths, 2001). Instead, another model proposed that auditory pathways could be segregated by their modes of auditory processing, such that a dorsal pathway extracts the message or melody from sound, whereas the ventral pathway identifies the speaker or instrument by its timbre (Zatorre et al., 2002b).

The independence of single frequency and harmonic processing is also critically important for the separation of auditory objects (e.g., Yost, 2007), because objects can be conceived as particular correlations of several frequency bands (Nelken et al., 2014). Moreover, the non-linear analysis of physical stimuli in the cochlea can result in internally generated new harmonics produced by the auditory system itself (Pickles, 1988). These facts demonstrate the inadequacy of the idea that the primary auditory processes sound in a Fourier-like manner.

Notably, the relation between the three auditory cortex regions (i.e., A1, A2, and A3) changed very much in the course of human evolution. While the primary auditory cortex in humans is slightly smaller than in macaques, the human belt and parabelt areas are almost 10 times larger (Angulo-Perkins and Concha, 2014). Another interesting fact is that the origin of auditory cortical input is mostly top-down. This is true even for A1, as only 23% of neurons projecting to A1 are of purely acoustic subcortical (i.e., thalamic) origin, while 66% are cortical neurons, most of them being localized at higher levels of the auditory system. Therefore, one cannot speak about feature analysis at the A1 level. Rather, stimulus representation in the auditory cortex is task-specific, i.e., “spatio-temporal activation patterns of neuronal ensembles in AC, passively generated by a given stimulus and basically reflecting all features of a stimulus, can be modified according to the context and the procedural and cognitive demands of a listening task, i.e., also reflect semantic aspects of a stimulus” (Scheich et al., 2007, p. 214).

As receptive fields of cortical neurons can flexibly adjust to the auditory task, the tonotopy of A1 should not be overvalued. Many A1 neurons in most investigated mammalian species respond to several frequencies (for primates, see, e.g., Sadagopan and Wang, 2009), and even those with a single-frequency peak do not respond to individual components of harmonic tones that are outside of its tone-derived frequency response area (Wang and Walker, 2012). This suggests that frequency-driven responses can be harmonically modulated. While the relatively few axons from the geniculate nucleus of the thalamus frequently end at cell bodies or basal dendrites, the big portion of the top-down cortical input comes to apical dendrites, thus creating a “context” modulating responsivity to specific factors. The relation between top-down and bottom-up input in higher-order areas is even more shifted toward the former. Together, these data support the view that the purpose of the auditory cortex in higher animals (mainly investigated in monkeys) is not only sensory analysis but also the adjustment to the auditory environment and identification of auditory objects (Yost, 2007; Reybrouck and Brattico, 2015).

### Human Studies

As cellular mechanisms of music perception at subcortical and cortical levels cannot be studied directly in humans, the neural characteristics of music processing have mostly been investigated using event-related brain responses measured with the EEG and the magnetoencephalogram (MEG), or by assessing the blood oxygenation (BOLD) response to auditory stimulation with functional magnetic resonance imaging (fMRI). The latter, for example, revealed that optimized auditory processing of rhythm and frequency is associated with a relative hemispheric advantage, with the left auditory cortex being more sensitive to temporal characteristics of auditory cues (i.e., more prevalent in speech production) and the right auditory cortex being better for decoding pitch and harmony content of acoustic stimuli, which is emphasized in music (Zatorre et al., 2002a). Given the huge difference in the methodological precision (each EEG, MEG, or fMRI recording encompasses the activity of many thousands of neurons, compared with single cell recordings in animals), however, one may even be surprised how similar are the conclusions of human and animal experiments.

The arrival of auditory input at the cortex in humans is manifested in ERPs by the obligatory (exogenous) primary complex P1–N1 with the latencies of about 50 ms and 100–120 ms for P1 and N1, respectively. Processing of stimulus deviation is reflected in an endogenous ERP component mismatch negativity (MMN: Näätänen, 1995) that attains its peak around 200 ms post stimulus. MEG data show that at least a large portion of the MMN is generated in the auditory cortex. An important property of the MMN is that its generators do not require active attention. Even though attention to stimuli can increase MMN amplitude (e.g., Erlbeck et al., 2014), other ERP components (which can mask the MMN) are increased to a much larger extent; therefore, it is practically better to record the MMN in a condition in which the subject’s attention is caught by some other activity such as reading a book or looking at a movie. Higher-order music processing can be manifested in an early right anterior negativity (ERAN), an ERP component of frontal origin (for review, see Koelsch, 2014), or in two late components, N400 and P600, with the latencies of about 400 and 600 ms, respectively. These components, however, are much more attention-dependent than the MMN.

For a long time, the MMN was studied in response to rather simple stimulus deviations such as deviations in pitch (e.g., 800 Hz–800 Hz–800 Hz–800 Hz–600 Hz), intensity (e.g., 80 dB–80 dB–80 dB–80 dB–65 dB), or tone duration (e.g., 50 ms–50 ms–50 ms–50 ms–30 ms). Later studies showed, however, that the MMN also responds to much more complex pattern changes in the auditory stream (e.g., Tervaniemi et al., 1994). Thus, the repetition of a short sequence like AAB results in an MMN after omission of the last tone (AA_), reversal (ABA), or even repetition of the same tone (AAA). Moreover, MMN mechanisms are also sensitive to some level of abstraction. This is shown in an experiment in which standard (repeated) stimuli were ascendant pairs combining five different chords (AB, CD, AC, BE, etc.). Two kinds of rare deviants were either descendent pairs (DA, CB, etc.), or repetitions (AA, DD, etc.). Both kinds of deviants elicited a strong MMN (Tervaniemi et al., 2001).

Dipole localization using MEG indicates that the generator of the MMN to chords in the STG is located more medial than the MMN generator for sine tones. However, stimulus complexity is not the only factor affecting the generator structures, as demonstrated by experiments in which the magnetic counterpart of the electric MMN was compared between phoneme change and chord change of the same acoustic complexity. The source of the “musical” MMN was located superior to the source of the “phonetic” MMN. Moreover, the former was lateralized to the right side, while the latter was symmetrical. Importantly, the generator of the component P1 was identical for all stimuli of comparable complexity regardless of their origin. Apparently the mechanism of the MMN is the first processing stage at which music-specific analysis of auditory stimuli begins (Angulo-Perkins and Concha, 2014).

In support of animal data presented above, indicating a strong independence of processing of harmonic tones compared to that of single sine frequencies, MMN data indicate that also in humans pitch deviations of chords result in a larger MMN than comparable deviations of pure tones (Tervaniemi et al., 2000). By successfully replicating this MMN paradigm in a large sample of DoC patients, our group demonstrated that the MMN to harmonic tones not only led to a larger amplitude as shown before but also to a higher frequency of occurrence than the MMN to sine tones (Kotchoubey et al., 2003). About a half of the patients who did not have an MMN to simple sine tones exhibited, however, an MMN to harmonic tones. The MMN seems to be present in about 30–60% of all patients with acute or chronic DoC (Kotchoubey, 2015). In acute coma it belongs to the most reliable predictors of further awakening (meta-analytic review of Daltrozzo et al., 2007), and there is also evidence of its predictive meaning in chronic DoC (Kotchoubey et al., 2005). In order to evaluate the effectiveness of music therapy in chronic DoC, the habitual assessment of MMN to complex tones could help developing a potential outcome predictor.

Other ERP components, later than the MMN, occur with a lower frequency in DoC, but confirm that the auditory system of many DoC patients remains flexible enough to process stimuli of very high complexity (Kotchoubey, 2015). Thus the attention-dependent component P3 in these patients responds, like the MMN, much better to harmonic stimuli than to sine tones (Kotchoubey et al., 2001). ERP responses to complex violations in rhythmic sound sequences have recently been demonstrated in 10 of 24 patients in deep post-anoxic coma who were additionally sedated (Tzovara et al., 2015).

Key messages:

• Auditory processing is related to one of the most basic processes underlying all higher forms of life, i.e., the processing of environmental events in their temporal sequence.
• The auditory cortex entails specialized regions for the processing of complex sounds and their components. Auditory scene analysis and the identification of auditory objects is an important task of the auditory cortex, which can result in clinically important dissociations between disorders that entail the processing of simpler versus more complex sounds.
• Consistent responses to chords and to changes in harmonic patterns have also been observed in DoC cases where cortical responses to sine tones could not be recorded. We therefore suggest complex sounds for auditory stimulation in DoC as a rule.
• Non-responsiveness to simple sounds is no reason to withdraw from musical therapy!

## Higher-Level Auditory Processing

### Segregation and Integration

Beyond basic aspects of sound processing, music perception represent a highly complex process that involves the segregation and integration of various different acoustic elements such as melody, harmony, pitch, rhythm, and timbre, which engage networks that are not only implicated in auditory but also in syntactic and visual processing (Schmithorst, 2005). In fact, both music and language engage partly overlapping (Liegeois-Chauvel et al., 1998; Buchsbaum et al., 2001; Koelsch and Siebel, 2005; Koelsch, 2006; Chang et al., 2010; Schön et al., 2010; Patel, 2011) as well as domain-specific subcortical and cortical structures (Belin et al., 2000; Tervaniemi et al., 2001; Zatorre et al., 2002a; Zatorre and Gandour, 2008).

Sound perception first requires the extraction of auditory features in the brain stem, the thalamus, and the auditory cortex (Koelsch and Siebel, 2005), leading to auditory percepts of *pitch-height and pitch-chroma, rhythm, and intensity*. However, the lower-level frequencies related to the temporal organization of music may also be processed independently from melodic intervals (Peretz and Zatorre, 2005), engaging additionally pre- and supplementary motor areas, the basal ganglia, and the cerebellum (Grahn and Brett, 2007; Thaut et al., 2009). This integration of sequentially ordered acoustic elements on longer time-scales is a highly demanding task that requires the structuring (e.g., separation or grouping) of musical elements, leading to a cognitive representation of acoustic objects based on Gestalt principles (Darwin, 2008; Ono et al., 2015). The cognitive involvement of musical pattern processing is evident from the joint activation of auditory association cortices with pre-frontal regions in the brain (Griffiths, 2001).

All basic forms of learning, some of which presented even in the simplest animals like worms, necessarily involve the ability to perceive events in their temporal order. Thus habituation results from perceiving one and the same stimulus as repeating; classical (Pavlovian) conditioning is based on the perception that one stimulus (CS) consistently precedes another one (UCS); and so on. The perception of sequential events is essential to all higher forms of life, because it allows for the timely preparation of appropriate responses. The steady anticipation of consecutively presented information units therefore relates music to one of the most fundamental necessities of life, the predictability of events in their temporal succession (e.g., Francois and Schön, 2014; Wang, 2015). Events that are out of rhythm are unpredictable.

The sequential ordering of individual pitches also leads to the perception of melody, whereas their vertical ordering leads to the perception of harmony. To achieve perceptual coherence, a rule-based hierarchical organization of acoustic inputs is therefore elemental for determining how tones may be combined to form chords, how chords may be combined to form harmonic progressions, and how they are all united within a metric framework. This process of hierarchical structuring and temporal ordering of acoustic objects is indeed a shared feature in the syntactic organization of both music and speech.

### Musical Syntax and Semantics

Syntax in music (just as in language), “refers to the principles governing the combination of discrete structural elements into sequences” (Patel, 2008, p. 241), with independent (yet interrelated) principles for melody, harmony, and rhythm. Musical syntax has been most thoroughly investigated with respect to harmony (e.g., Koelsch, 2012), as syntactic perception of harmonic dissonance and consonance depends crucially on the functional relationships of preceding and subsequent chords (or tones). As outlined above, these percepts build on expectancies based on previously acquired long-term knowledge and thus trigger distinct responses in the brain when they are violated.

An early study with musicians by Janata (1995) demonstrated that the violation of expectancy in the final chord of a chord sequence elicits larger P3 peaks as a function of the degree of violation, thus reflecting both attentional (P3a; 310 ms latency) and decisional (P3b; 450 ms latency) processes. Another study (Patel et al., 1998), reported that incongruences in both language and music syntactic would elicit a parieto-temporal P600, which had been associated with language processing, suggesting that this ERP component reflects more general processes of structural acoustic integration across domains. Likewise, some kinds of syntactic violations in language may elicit a specific negative component in the ERP with a latency about 200–300 ms and a maximum over the left frontal cortex, the so-called early left anterior negativity (ELAN). Beginning with a first study by Koelsch et al. (2000), a comparable syntactic violation in music was found to result in a quite similar ERP component over the right frontal cortex: the ERAN (Koelsch et al., 2001; Koelsch and Jentschke, 2010; Koelsch, 2012). Accordingly, the ERAN reflected “a disruption of musical structure building, the violation of a local prediction based on musical expectancy formation, and acoustic deviance” (Koelsch, 2012, p. 111). A later negative component around 500–550 ms (N5) was also observed over frontal regions following the ERAN, but was rather associated with musical meaning (Poulin-Charronnat et al., 2006, see below). Other, simpler kinds of syntactic violations resulted mainly in a late positive parietal complex rather than an early frontal negativity for both language (e.g., Osterhout, 1995) and music (e.g., Besson and Faïta, 1995), although studies on melodic syntactic violations also reported a frontal ERP response with a slope emerging around 100 ms and peaking around 120–180 ms that resembled the ERAN in harmonic violation paradigms (Brattico et al., 2006; Koelsch and Jentschke, 2010).

A conceptual similarity between music and speech perception is also reflected in the dynamics of the N400 ERP component (e.g., Patel, 2003; Kotchoubey, 2006; Daltrozzo and Schön, 2009a,b). Like the N5, the N400 has been attributed to musical meaning rather than syntax, contributing to the subjective interpretation of musical information, which involves affective processing. Koelsch (2012) used the term musical semantics to account for the different dimensions of extra-musical, intra-musical, and musicogenic meaning. Extra-musical meaning can be derived from musical sign qualities by making reference to the extra-musical world, such as the imitation of naturally occurring sounds (e.g., the river Rhine in Wagner’s “Rheingold” prelude), the psychological state of a protagonist (e.g., in the pranks of Richard Strauss’s “Till Eulenspiegel”), or arbitrary symbolic associations (e.g., national anthems). Intra-musical meaning in turn refers to the interpretation of structural relations between musical elements, whereas musicogenic meaning describes the experience of emotional, physical, or personal effects of music, which are evoked within the listener.

Several studies have demonstrated that the representation of extra-musical meaning can be related to the N400, which is thought to reflect to the processing of meaning, for example when the content of target words in a semantic priming paradigm is meaningfully unrelated to the content of preceding musical excerpts (Koelsch et al., 2004). The N400 seems to be generated in the posterior temporal lobe, in close vicinity to regions that also process speech related semantics (Lau et al., 2008) and non-verbal vocalization (Belin et al., 2000; Kriegstein and Giraud, 2004). The notion that the N400 processes meaning from musical information has been confirmed in recent studies (Goerlich et al., 2011), where the N400 was triggered when the affective valence of word primes did not match the valence of musical or prosodic stimuli. Intra-musical meaning, in contrast, seems to be reflected by the N500 (or N5). As indicated above, the N5 follows the ERAN elicited by the perception of harmonic incongruence. However, the N5 does not just represent a function of incongruity in harmonic progressions but is rather modulated by the harmonic integration and contextual information in music that is not related to an extra-musical reference (Steinbeis and Koelsch, 2008). Lastly, musicogenic meaning may emerge from emotions evoked by the musical stimulus, which can also be associated with corresponding personal memories (see music evoked emotions below).

Although we do not know about any direct effects of music listening on language comprehension or other verbal functions in DoC patients, such effects have been demonstrated in other clinical populations. Music training has been used in language disorders (Daltrozzo et al., 2013) and the rehabilitation of aphasia patients, which led to increased structural integrity of white-matter tracts between fronto-temporal regions involved in language processing (Schlaug et al., 2010; Marchina et al., 2011). Also perceptual treatments have shown strong effects, including increased gray-matter volume after passive musical and verbal stimulation in stroke patients (Särkämö et al., 2014a). In this study, long-term changes (6-month follow up) were found in the orbitofrontal cortex, anterior cingulate cortex, ventral striatum, fusiform gyrus, insula, and superior frontal gyrus (SFG) areas after patients listened regularly to their preferred music. Changes in frontolimbic cortex moreover correlated with the improvement of verbal memory, speech and focused attention. Thus the SFG and the anterior cingulated cortex (ACC) appear to be important structures that mediate between music processing and cognition.

Key messages:

• Music and language both work with temporal features of stimulation. The two domains are implemented in partially overlapping, partially analogous morphological and functional mechanisms. Successful therapeutic interventions in one of these domains can result in significant improvement in the other one as well.
• We propose that the distinct ERP components associated with the neural difference in the processing of musical syntax and musical semantics (i.e., extra-musical, intra-musical, and musicogenic meaning) may prove useful for the detection of disparate cognitive processes during music perception in DoC.

## Implications for Multisensory Stimulation

Although both music and speech perception are based on auditory scene analysis (Janata, 2014), perceptual modalities should not be treated as independent entities but rather considered in the context of simultaneous multisensory integration, which explains why somatosensory and visual feedback can significantly modulate auditory perception (Wu et al., 2014). In the same vein, the close connection between production and perception in music and speech tightly links auditory and somatosensory modalities. During production, we compare acoustic feedback with the intended sound to adjust motor commands, yet we simultaneously develop corresponding somatosensory representations related to inputs from cutaneous and muscle receptors (Ito and Ostry, 2010; Simonyan and Horwitz, 2011). Based on Hebbian learning mechanisms (Hebb, 1949), this simultaneous co-activation of perceptual and motor systems leads to the phenomenon of cross-modal plasticity, which manifests as mutual facilitation of neural activity and explains altered perception in one modality when the expected sensory feedback of another modality is not in register (Gick and Derrick, 2009). For example, stretching the facial skin during listening to words alters the subjective perception of auditory feedback (Ito et al., 2009). Conversely, the manipulation of auditory feedback during speech can also alter somatosensory orofacial perception (Ito and Ostry, 2012). Champoux et al. (2011) demonstrated that amplitude modulation of auditory feedback during speech production can even induce distinct laryngeal and labial sensations that are not a mechanic consequence of the motor task, whereas Schürmann et al. (2006) showed that vibrotactile stimulation helps auditory perception in both healthy and hearing impaired subjects.

As a rule, mutual perceptually facilitating effects are stronger when co-activation has been learned over a longer period, as shown in the example of trained musicians. In a study from Christo Pantev’s lab (Schulz et al., 2003), professional trumpet players and non-musicians received auditory (i.e., trumpet sound) and somatosensory (i.e., lip) stimulation, presented either alone or in combination. Results showed that the combined stimulation yielded significantly larger responses in MEG source waveforms in musicians than in non-musicians, suggesting that the stronger experience in task-dependent co-stimulation of somatosensory and auditory feedback facilitates their cross-modal functional processing in musicians (Pantev et al., 2003). Similar effects have been described for audio-visual processing of music, corresponding to an increased N400 response when the two modalities were incongruent. Studies in the speech domain furthermore suggest that accurate corrective vocal-motor responses to somatosensory and auditory perturbation exist in both modalities (Lametti et al., 2012), although somatosensory feedback seems to gain importance as experience increases in trained singers (Kleber et al., 2010, 2013).

The logic behind cross-modal plasticity in the context of DoC is related to the idea that simultaneously stimulating functionally corresponding auditory and somatosensory modalities could potentially boost (i.e., facilitate) the neural responses in both systems. Although there is no large-size statistical data about the frequency of somatosensory disorders in DoC, somatosensory EP (SSEP) are standardly recorded in most hospitals for neurological rehabilitation. In fact, the functionality of somatosensory pathways has been successfully used to predict the long-term outcome of these disorders (de Tommaso et al., 2015; Li et al., 2015). Therefore, we suggest that the somatosensory system can be explored by means of neurophysiological techniques.

The idea of using more than one sensory modality for interacting or stimulating DoC patients is not new. In fact, “basal” multisensory (i.e., visual, auditory, tactile, gustatory, and olfactory) stimulation has been used as a therapeutic intervention and represents a standard procedure in many German intensive care and early rehabilitation facilities (Menke, 2006). However, multisensory stimulation in DoC patients is not standardized and the therapeutic use of multisensory stimulation has not been well documented (Rollnik and Altenmüller, 2014). Moreover, the concurrent stimulation of individual sensory modalities may be functionally unrelated and thus not trigger a facilitating effect, which could account for the lack of reliable evidence to support the effectiveness of multisensory stimulation programs in patients in coma or the VS (Lombardi et al., 2002). We therefore propose to apply multisensory stimulation only in a functionally related way, for example with concurrent orofacial-tactile and corresponding auditory stimulation associated with song or speech production. This might increase chances to enhance the potential of multisensory stimulation for the detection of diagnostic ERP components in DoC and/or to facilitate therapeutic processes.

A similar line of thought follows the tight coupling between perception and action when we synchronize our body movements to an external rhythm even without being aware of it. Timing is extremely important for movement, which can be facilitated by music perception via activation of distinct cerebellar-cortical networks involved with movements control (Thaut et al., 2009). Indeed rhythm production and perception engages similar brain regions including the supplementary motor area (i.e., involved in motor sequencing), the cerebellum (i.e., involved in timing), and the pre-motor cortex (Chen et al., 2008a). In musicians, activity in pre-motor cortex has been linked to the rhythm difficulty, suggesting that also working memory contributes to the organization and decomposition of acoustic temporal structures (Chen et al., 2008b). The involvement of pre-frontal and temporal regions during auditory rhythm stimulation has been confirmed with both electrophysiological (direct current; Kuck et al., 2003) and PET data (Janata, 2014). The latter study found furthermore common activation patterns for rhythm, meter, and tempo within frontal, pre-frontal, temporal, cingulate, parietal, and cerebellar regions. Not surprisingly, auditory rhythmic stimulation has been successfully used to facilitate motor acts in both healthy subjects and in neural rehabilitation (Molinari et al., 2003; Chen et al., 2006), since musical rhythms activate a network that is otherwise engaged by motor production and that can be distinguished from melodic processing (Bengtsson and Ullen, 2005).

Key messages:

• Multisensory stimulation in DoC is suggested to take into account the potentially facilitating effects of cross-modal plasticity as a result of functionally corresponding processes during production and perception in well-trained motor tasks (e.g., speech or song).
• The strong link between musical rhythm and motor behavior might be useful for testing motor related responses to rhythmic auditory stimulation as a complementary approach to the testing of syntactic (melodic/harmonic) processing in the brain of DoC patients.

## Therapeutic Effects of Musical Stimulation

### Cognitive Effects

Music production is a uniquely rich multisensory experience. The development of musical skills enhances not only the cognitive, sensorimotor, and perceptual abilities but also changes corresponding motor, sensory, and multimodal representations in the brain (Herholz and Zatorre, 2012). Although these changes are particularly apparent in trained musicians, available clinical studies indicate that musical stimulation and musical training can also have beneficial effects for the rehabilitation of higher-order cognitive functions, e.g., on autobiographical memory in Alzheimer’s patients (Irish et al., 2006; El Haj et al., 2012; García et al., 2012) and other kinds of dementia (Foster and Valentine, 2001). Irish et al. (2006) found that participants with mild Alzheimer’s disease were recalling significantly more life events when listening to Vivaldi’s “The Spring” compared to a silence condition, whereas the same effect was even higher with patients’ self-chosen music (El Haj et al., 2012).

Possible mechanisms underlying the effect of musical stimulation on cognitive functions in patients with severe neurological disorders may be associated with neuroplasticity and neurogenesis in brain regions that are activated by music. Neuroplasticity may result in healthy brain areas compensating the disordered functions of injured areas and/or may increase the rate of neurogenesis and gray matter volume. The effect of music on neuroplasticity has been demonstrated in several studies (Stewart et al., 2003; Rickard et al., 2005; Pantev and Herholz, 2011; Herholz and Zatorre, 2012; Särkämö and Soto, 2012) and appears to be, at least partly, mediated by the production of the neurotrophin BDNF (brain-derived neurotrophic factor) in the hippocampus, which is increased in music-rich environments (Angelucci et al., 2007; Marzban et al., 2011) and involved in processes of memory formation and learning.

Another explanation for the effects of music on cognition involves the ACC and its product, the frontal midline theta rhythm, which is crucially important for emotional and cognitive processes (Bush et al., 2000). The frontal midline theta (fm-theta) is involved in working memory (Klimesch, 1997, 1999; Doppelmayr et al., 2000), episodic memory (Klimesch, 1997; von Stein and Sarnthein, 2000), emotional processing (Aftanas and Golocheikine, 2001), cognitive control (Gruendler et al., 2011; Cavanagh and Frank, 2014), and executive functioning (Miyake et al., 2000; Fisk and Sharp, 2004). In healthy subjects, ACC activation was found to correlate with pleasure responses to music (Blood and Zatorre, 2001; Baumgartner et al., 2006). Accordingly, the spectral power of the fm-theta is increased during listening preferred pleasant music in contrast to unpleasant one (Sammler et al., 2007). Interestingly, the only study that investigated the cognitive correlates of music perception in DoC patients replicated this effect (O’Kelly et al., 2013). In this study, the information about personal music preferences in patients was obtained from their close relatives, while for control participants this information was obtained directly. Listening to preferred songs has increased the power of the fm-theta in both groups.

To avoid superficial optimism, it should be said that the effects of music on cognition could critically depend on the amount of training. Probably in this case the rule of “the more the better” works. Särkämö et al. (2014b) attained significant effects of musical stimulation after 10 weeks of intensive training in 29 patients with dementia, which included not only passive listening to music but also conversations in small groups about the music-evoked emotions, thoughts and memories. Moreover, participants also performed homework assignments dedicated to listening their favorite music, while their caregivers organized the music intervention sessions. Beneficial effects at the 9-month follow-up involved a positive correlation between participants’ mood, working memory performance and the frequency of music sessions. Together, these findings indicate that music therapy and stimulation can have significant effects on cognitive and emotional aspects, whereas the intensity of music intervention can play a key role for producing long-lasting and stable structural and functional changes in the brain.

Key messages:

• Passive listening to preferred music over longer time-periods might particularly enhance processes related to memory and cognition in DoC.
• Changes in fm-theta amplitudes during listening pleasant music could indicate emotional and cognitive responses.
• More intensive music therapy interventions might provide better therapeutic results.

### Emotional Effects

The putative association between music stimulation and cognitive improvement in DoC patients might also be mediated by positive music-evoked emotions. These positive emotions can be associated with activation of the reward system of the brain and related dopamine release. At the same time, dopamine levels can be directly related to working memory, cognitive control, and attention (Nieoullon, 2002; Cools and D’Esposito, 2011). Pharmacological studies have shown that the increase of dopamine level improves performance in working memory and executive functions in both healthy subjects (Mehta and Riedel, 2006) and patients with traumatic brain injuries (Bales et al., 2009).

Music is a potent stimulator for a wide range of basic and complex emotions associated with changes in physiological arousal, subjective feeling, and motor expression (Koelsch et al., 2006, 2008; Grewe et al., 2007a,b). The reward value of music is moreover reflected in the classic reward circuitry of the brain (Zatorre, 2015), which entails dopaminergic mesolimbic pathways including the ventral tegmental area, the striatum (dorsal: nucleus accumbens; ventral: the head of the caudate nucleus), the ventromedial and orbitofrontal cortices, the amygdala, and the insula (Berridge and Kringelbach, 2013). These regions are traditionally associated with primary and secondary rewards, yet pleasurable music is also able to activate this system (Koelsch, 2014). For example, dopamine release in response to music stimulation accompanied by pleasurable emotional reactions has been reported in a study by Salimpoor et al. (2011).

The positive effects of music on emotional states (and correspondingly cognitive processing) may be related to acoustic features of music but have also been attributed to familiarity, as the subjective liking of music can be directly correlated with the familiarity of the piece (Peretz et al., 1998; Schellenberg et al., 2008). Listening to familiar versus unfamiliar music yields higher activity in the limbic system and the orbitofrontal cortex (Pereira et al., 2011), which is in accord with data demonstrating a correlation between music-elicited positive emotions and orbitofrontal activation (Menon and Levitin, 2005).

Familiarity implies the anticipation of a pleasurable musical passage, in line with the difference between anticipation and actual experience that has been found by Salimpoor et al. (2011). That is, activation in dopaminergic areas peaked in the dorsal striatum seconds before the maximum pleasure was experienced, related to the number of chill experiences, whereas activation in the ventral striatum was associated with the emotional intensity at the moment of the peak pleasure experience. Yet also novel (i.e., unfamiliar) pieces of music can trigger responses in the dorsal striatum when their reward values are high (Salimpoor et al., 2013), which was taken as further evidence that temporal (i.e., musical structural) predictions may also be involved in the emotional experience of music. On the other hand, striatal connectivity with auditory cortex that increased as a function of reward value suggests that previous memory formation could affect expectations related to emotional experience in music. Individual differences in memory formation could therefore modulate both the anticipation of intra-musical meaning (i.e., based on statistical leaning of functional relationships between consecutive musical elements) and the allocation of personal “musicogenic” meaning to a musical sequence (i.e., based on personal relevance). In addition, episodic memory and musical valence are closely interrelated, such that musical pieces with a positive association are also better remembered (Eschrich et al., 2008).

Särkämö and Soto (2012) suggested that the effects of music on working memory and attention performance, which they observed in stroke patients, were partly mediated by dopamine increase related to positive emotion. This idea is supported by the fact that depression and confusion were inversely correlated with verbal memory performance after music therapy. In another study including patients with visual neglect, the same research team (Soto et al., 2009) showed that listening to pleasant music enhanced awareness to contralesional targets.

Interestingly, brain injuries leading to DoC are often related to widespread damage of dopaminergic system axons and a reduced level of dopamine in the cerebrospinal fluid (Meythaler et al., 2002). There is even a hypothesis that DoC are mainly caused by destruction in the dopamine system (Hayashi et al., 2004), whereas restoration of the normal regulation of dopamine level has a positive effect on cognitive recovery in DoC patients. In several studies, levodopa (a precursor of dopamine) not only improved motor functions of DoC patients but also resulted in positive changes of their consciousness (Haig and Ruess, 1990; Matsuda et al., 2003, 2005; Krimchansky et al., 2004; Ugoya and Akinyemi, 2010). Moreover, the well-known placebo-controlled randomized study of traumatic DoC patients (Giacino et al., 2012) revealed a significant effect of the indirect dopamine agonist Amantadine.

A recent study (Castro et al., 2015) demonstrated the aforementioned relationship between music, familiarity, and cognition in a sample of DoC patients. The study included the presentation of the subjects’ own first name (SON) as a deviant stimulus among other first names as standard stimuli. Listening to excerpts from the patient’s preferred music increased the amplitude of ERP components N2 and/or P3 to SON in seven of 13 patients. These seven patients showed a favorable outcome after 6 months following the experiment. The other six patients who did not show any response to the SON remained in the same state or died 6 months later. The existence of music-evoked emotions in DoC might therefore even have a predictive value in DoC and perhaps also the potential to re-activate memory traces associated with musical emotions.

Key messages:

• DoC can be related to damage of the dopaminergic system. Emotionally pleasurable music in turn can activate the dopaminergic system by inducing changes in the limbic system associated with the reward value of music, which could have beneficial effects on consciousness in DoC.
• Music therapy and musically induced positive intra-musical and musicogenic emotions might furthermore stimulate cognitive processes and personal memory activation.
• A hypothesis worth testing is that ERP components, such as the N2 and/or P3 in response to preferred music as well as changes in time-frequency theta amplitudes over frontal midline regions in the EEG, might predict the outcome of DoC in response to emotionally pleasurable music.

### Stress Reduction

Influence of stress and the related cortisol level on cognitive functions was shown in numerous studies with healthy participants, where the increased level of cortisol had a negative impact on executive functions, declarative memory, working memory, and language comprehension (McEwen and Sapolsky, 1995; Lupien et al., 1997; Lee et al., 2007). Factors mediating the negative impact of chronic stress are supposed to be dendritic atrophy and synaptic loss in the hippocampus and the prefrontal cortex as well as the decrease of the rate of neurogenesis in the hippocampus (Radley and Morrison, 2005). Chronic stress can also cause changes in the dopaminergic system, reducing dopamine levels in the prefrontal cortex (Mizoguchi et al., 2002), and negatively affect the immune system (Segerstrom and Miller, 2004).

Several studies have emphasized the stress-reducing value of daily music listening, with positive effects being observed on subjective, physiological, and endocrinological parameters (Linnemann et al., 2015). Even short-term exposure to musical stimulation consistently decreases cortisol levels of healthy subjects (for systematic review, see Fancourt et al., 2014) and this effect was particularly large when participants had self-selected the music (e.g., in patients undergoing surgery; Leardi et al., 2007). Moreover, there is also evidence for positive effects of music on the immune system, as indicated by several parameters at cytokine, leukocytes and immunoglobulins levels (Fancourt et al., 2014).

Convincing evidence suggests that traumatic brain injury, stroke, and other frequent neuropathological factors can induce stress reactions in both short-term (Franceschini et al., 2001; Prasanna et al., 2015) and long-term (Sojka et al., 2006; Marina et al., 2015) perspectives. These findings suggest that DoC of traumatic or non-traumatic etiology may also be accompanied by chronic stress, although the available data are inconsistent. While Vogel et al. (1990) obtained an increased level of cortisol in VS using 24 h monitoring, Munno et al. (1998) found a lower cortisol level in VS patients and in a group of exit-VS patients who had been conscious for more than 6 months in comparison with normal parameters. Another study of VS patients in a long-term-care facility (mean disease duration 6.2 ± 5.1 years) did not reveal any significant differences from a control group (Oppl et al., 2014). A case study reported a VS patient whose level of consciousness improved after injections of autologous activated immune cells (Fellerhoff et al., 2012).

Key message:

• Music has the potential to enhance cognitive functions in DoC through a decrease of stress and a related drop of cortisol level together with activation of the immune system.

## Conclusion

Direct evidence for positive effects of music therapy interventions on cognitive functions in DoC is still very scarce. In this paper we summarized a theoretical justification for the idea that properly organized music stimulation programs can indeed lead to the suggested beneficial effects. At the low-level organization of the (primary and secondary) sensory cortical areas, the auditory modality reveals its particular potential for presenting specific stimulation that combines sufficient complexity with the availability for severely brain-damaged patients. In this context, we strongly suggest the use of complex sounds rather than sine-tones in DoC. Cognitive mechanisms would capitalize the specific psychological and neurophysiological affinity between music and speech processing, based on the great similarity between these two domains of human culture. This entails the identification of auditory objects, which can result in clinically important dissociations between disorders based on the processing of musical syntax and meaning, which are reflected by changes in corresponding ERP components. The neuroplastic associations with music may furthermore lead to functional improvement of memory and attention beyond the language domain, whereas multisensory stimulation based on previously acquired cross-modal plasticity may facilitate electrophysiological responses as well as functional improvement. Moreover, musically stimulated rhythmic processes in the nervous system could serve as a starting point for rehabilitation. A completely different mechanism mediating the hypothesized positive effects of music in DoC runs through music-evoked emotions, which have the potential to activate the dopaminergic system and may thus lead to a suppression of the stress response system. The diagnostic value of musically evoked emotions includes ERP components such as the N2 and/or P3 as well as changes in time-frequency theta amplitudes over frontal midline regions in the EEG. However, more research is needed to address the ecological validity of these suggestions and thus to come to more conclusive results in this patients group, even though the organization and performance of such studies is highly demanding.

### Conflict of Interest Statement

The authors declare that the research was conducted in the absence of any commercial or financial relationships that could be construed as a potential conflict of interest.
